# Impact of declining oxygen conditions on metal(loid) release from partially oxidized waste rock

**DOI:** 10.1007/s11356-019-05115-z

**Published:** 2019-05-18

**Authors:** Hanna Kaasalainen, Paula Lundberg, Thomas Aiglsperger, Lena Alakangas

**Affiliations:** 1 Applied Geochemistry, Luleå University of Technology, 971 87 Lulea, Sweden; 2 Present Address: Sweco Environment, Västra Varvsgatan 11, 972 36 Lulea, Sweden

**Keywords:** Mine closure, Waste rock, Mine waste, Metal mobility, Water quality, Remediation

## Abstract

The best available technology for preventing the formation of acid drainage water from the sulfidic waste rock at mine closure aims to limit the oxygen access to the waste. There is, however, a concern that contaminants associated with secondary minerals become remobilized due to changing environmental conditions. Metal(loid) mobility from partially oxidized sulfidic waste rock under declining and limited oxygen conditions was studied in unsaturated column experiments. The concentrations of sulfate and metal(loid)s peaked coincidently with declining oxygen conditions from 100 to < 5 sat-% and to a lesser extent following a further decrease in the oxygen level during the experiment. However, the peak concentrations only lasted for a short time and were lower or in the similar concentration range as in the leachate from a reference column leached under atmospheric conditions. Despite the acid pH (~ 3), the overall quality of the leachate formed under limited oxygen conditions clearly improved compared with atmospheric conditions. In particular, the release of As was two orders of magnitude lower, while cationic metals such as Fe, Cu, Mn, and Zn also decreased, although to a lesser extent. Decreased sulfide oxidation is considered the primary reason for the improved water quality under limited oxygen conditions. Another reason may be the immobility of Fe with the incorporation of metal(loid)s in Fe(III) minerals, in contrast to the expected mobilization of Fe. The peaking metal(loid) concentrations are probably due to remobilization from solid Fe(III)-sulfate phases, while the relatively high concentrations of Al, Mn, and Zn under limited oxygen conditions were due to release from the adsorbed/exchangeable fraction. Despite the peaking metal(loid) concentrations during declining oxygen conditions, it is clear that the primary remediation goal is to prevent further sulfide oxidation.

## Introduction

Oxidative weathering of sulfidic mine wastes releases acidity, sulfate, and metals to the environment, often resulting in the formation of acidic water rich in metal(loid)s and sulfate, known as acid mine drainage (AMD), which is one of the key environmental problems associated with mining activities. Rather than treating the formed AMD, the best available technology for the remediation of sulfidic mine wastes at mine closure is to prevent, or at least to minimize, the oxidation of sulfides and, thereby, the AMD formation (e.g., INAP 2018). A common remediation approach in case of waste rock is to apply a soil cover over the dump, serving to reduce water infiltration and oxygen diffusion to the waste, therefore promoting anoxic conditions in the waste deposit (Sahoo et al. 2013). Since the remediation of waste rock is typically not initiated before the decommissioning phase of the mine, the waste rock deposited in the dumps at the ground surface during operation may have been exposed to oxidation over several years or even decades prior to remediation. As a result, secondary minerals, such as jarosite (KFe_3_(OH)_6_(SO_4_)_2_), schwertmannite (Fe_8_O_8_(OH)_6_(SO_4_)·nH_2_O), ferrihydrite (nominally Fe_5_HO_8_·4H_2_O), and goethite (α-FeOOH), often collectively referred to as hydrous ferric oxides (HFO), may have formed and accumulated into the dumps (e.g., Bigham and Nordstrom 2000). Hydrous ferric oxides are typically characterized by low crystallinity and high specific surface area, making them very efficient metal(loid) scavengers (e.g., Webster et al. 1998; Bigham and Nordstrom 2000; Regenspurg and Pfeiffer 2004; Sidenko and Sherriff 2005). Thus, HFO often play a critical role in storing acidity and scavenging potentially harmful elements in the mine waste environment. However, HFO are only stable within a limited range of pH, redox conditions, and solution composition and tend to transform to more crystalline phases, such as goethite, with time (e.g., Bigham and Nordstrom 2000; Jönsson et al. 2005; Pedersen et al. 2005; Kumpulainen et al. 2008). Therefore, there is a concern that changed environmental conditions initiated upon remediation results in their dissolution with subsequent remobilization of the associated metal(loid)s.

Remediation of partially oxidized mine waste is often faced with challenges. For example, oxygen-limiting cover applied on partially oxidized, already acidic tailings was observed to be unable to prevent sulfide oxidation (Pabst et al. 2017), and elevated metal concentrations have been observed decades after subaqueous disposal of partially oxidized waste rock at several remediated mine sites in Sweden (Swedish Geological Survey (SGU) and Swedish Environmental Protection Agency (SEPA) 2017). One of the reasons for these observations has been suggested to be indirect sulfide oxidation by Fe(III) (SGU and SEPA 2017; Pabst et al. 2017). However, the underlying mechanisms, whether indeed involving reduction of Fe(III) or other mechanisms such as transformations of Fe(III)-containing minerals or release of metals from exchangeable sites, were not specifically addressed and therefore remain unresolved. Several studies on tailings and water-saturated waste rock have suggested that dissolution of secondary minerals and consequent mobilization of metal(loid)s are important contributors for water quality associated with partially oxidized sulfidic mine waste under laboratory and field conditions (e.g., Simms et al. 2000; Paktunc 2013; DeSisto et al. 2017). The application of these findings to unsaturated waste rock dumps is not straightforward due to the differences that exist in the oxygen and contaminant transport and sulfide oxidation processes in tailings and waste rock under water-saturated conditions and the unsaturated waste rock dumps. Waste rock dumps are characterized by much higher complexity arising from the large heterogeneity in particle size and often rock type, porosity compared with tailings deposits, and unsaturated flow conditions (e.g., Stockwell et al. 2006; Amos et al. 2015; Langman et al. 2015, 2017). There is clearly a need to better constrain metal(loid) leaching from the partially oxidized waste rock under changing chemical conditions, considering the expected increase in the waste rock production due to more advanced mining and ore processing techniques and the more stringent requirements regarding the environment in mine permitting and closure procedures.

Sweden is a country of active mining industry, and in the year 2016 alone, 62 Mtons of waste rock was produced by the mining activities (SGU 2017). A number of mine sites have been remediated during the last decades with variable success (SGU and SEPA 2017). Although there are many factors affecting the remediation and often the conditions before and after remediation have not been studied and documented in detail, remediation of partially oxidized sulfidic waste rock has shown particularly challenging. Here, we focus on assessing the impact of anoxic conditions on metal(loid) mobility associated with partially oxidized, highly sulfidic (ca. 20 wt% sulfide S) waste rock originating from an active open-pit Cu, Zn, Au, and Ag mine, Maurliden, operated by Boliden Mineral AB in northern Sweden. To this end, we studied the release of sulfur and metal(loid)s upon leaching of partially oxidized waste rock under declining and limited oxygen condition in unsaturated, free-draining column leaching experiments over a 15-month period and compared with leaching under atmospheric conditions. Special emphasis was given on As, Cu, Pb, and Zn, previously reported at high concentrations in the leachate from the waste rock and having a tendency to become sequestered into secondary minerals.

## Methods and materials

### Partially oxidized highly sulfidic waste rock

Waste rock used in the present study originates from a Zn, Au, and Ag mine, Maurliden, associated with volcanic-associated massive sulfide deposit in the Skellefte field, northern Sweden (Montelius 2005). Waste rock with very high sulfide content, ca. 50% pyrite, has been specifically selected from the mine for various types of experimentation, based on the S-contents determined in the field with the help of a handheld XRF (Olympus Innov-X systems, USA) (e.g., Alakangas et al. 2013; Nyström et al. 2017). Thus, it does not represent the bulk waste rock at the mine site. Previous studies indicate that waste rock mineralogy is dominated by pyrite and quartz, with traces of muscovite, chlorite, and calcite, as well as chalcopyrite, bournonite, sphalerite, and arsenopyrite (Nyström et al. 2017). The waste rock contains elevated concentrations of As, Hg, and Sb (Nyström et al. 2019).

Prior to its use in the present experiments, the waste rock had undergone ca. 6 years of weathering: 1 year in the field prior to its collection, followed by 5 years of leaching in four pilot-scale experiments under unsaturated or water-saturated conditions (Alakangas et al. 2013). Medium to coarse gravel–sized waste rock particles, coated with secondary precipitates of reddish–brown and brownish–red color characteristic for HFO, were hand-picked from the four pilot-scale experimental cells. Sampled waste rock was dried at room temperature, mixed, and sieved to desired particle size (passing 45.5-mm sieve size, > 12.5 mm). The particle size used in the experiment was governed by the availability of partially oxidized waste rock in the experimental cells, while the finest particle sizes were excluded to avoid clogging of the columns. The mixture was divided into four subsamples by coning and quartering. One subsample was reserved for geochemical and mineralogical characterization and three used in the column experiments. After termination of leaching experiments after ca. 15 months, samples of the waste rock were collected for selected geochemical and mineralogical analyses. Samples from the anoxic column were dried and stored in diffusion bags filled with Ar gas until the analyses.

#### Chemical composition

The waste rock was subjected to whole rock analyses, mineralogical characterization, and sequential chemical extractions. For determination of the whole rock composition, samples were pulverized and subjected to sodium peroxide fusion (SGS, Pittsburgh, USA) and a combination of four acid digestion (for Ag, Cd, Co, Cu, Li, Mo, Ni, Pb, Sc, Zn), aqua regia digestion (for volatile elements As, Hg, Sb), and lithium borate fusion (for remaining elements) (ALS Global). The digests were analyzed for major and trace elements by inductively coupled plasma atomic emission spectrometry (ICP-AES) and inductively coupled plasma mass spectrometry (ICP-MS) in both cases. Concentrations of F and Cl in the waste rock were determined by using ion selective electrodes (ALS Global). The sodium peroxide provides complete chemical analyses of resistant minerals without volatilization due to lower fusion temperature compared with lithium borate. However, except for Ni and W, the two analytical approaches showed an overall good agreement. Only the results from the sodium peroxide fusion (SGS), done on the same assays as the sequential extraction are reported here, with the exception of Na, F, Cl, S, and C that are based on ALS Global (Table 1). The forms of sulfur (S) and carbon (C) including carbonate-C, and carbonate-leachable and HCl-leachable S fractions were determined (ALS Global, methods C-GAS05, S-Gra06, S-Gra06a), along with total C and S with Leco furnace. Observe that sample pretreatment involved pulverization, but not all the elements and minerals in the waste rock are available for water–rock interactions.

#### Mineralogy

The mineralogy of primary and secondary mineral phases in the waste rock was studied via a multidiscipline approach:

(i) Optical microscopy of polished thin sections in transmitted and reflected light mode. Eight thin sections consisting of at least four waste rock particles each were prepared.
(ii) X-ray diffraction (XRD) of pulverized samples at the Centres Científics i Tecnológics at the Universitat de Barcelona (CCiT-UB) using a diffractometer PANalytical X’Pert PRO MPD Alpha1 and the following settings: *θ*/2*θ* Bragg–Brentano, radiation Kα1 of Cu (*λ* = 1.5406 Å), 45 kV–40 mA. Subsequent interpretation of the obtained diffractograms was done using XPert Highscore® software.
(iii) Raman spectroscopy using a Bruker Senterra equipped with an Olympus BXFM optical microscope at the Luleå University of Technology. Raman spectra were obtained using a 532-nm laser beam (*d* = 1 μm) at 0.2 mW with 45-s integration time and three repetitions.
(iv) Scanning electron microscopy and energy-dispersive spectroscopy (SEM–EDS) using a ZEISS Merlin HR-SEM at Luleå University of Technology. The conditions for measurements were 20-keV accelerating voltage and 1.1-nA beam current.

#### Sequential extraction

In order to gain quantitative information on the element distribution in primary and secondary mineral phases, and under which conditions the elements may become mobilized, the waste rock samples were also subjected to seven-step sequential extractions at the SGS laboratory (Pittsburgh, USA). The sequential extraction scheme by Dold (2003) is adapted to the specific secondary and primary mineralogy of mine tailings from Cu-sulfide ores but has been widely applied to other types of mine wastes as well. Here, only the first four steps were considered and compared with the whole rock composition from NaOH fusion:

(i) Step 1—de-ionized water leach for the water-soluble fraction, including dried pore water, metal salts, and gypsum
(ii) Step 2—ammonium acetate leach (pH 4.5) for the exchangeable fraction, but also dissolving carbonates and smectite
(iii) Step 3—cold oxalic acid leach (pH 3) in the dark to dissolve easily reducible Fe–Mn(–Al) (hydr)oxides and hydroxysulfates
(iv) Step 4—hot oxalate extraction (pH 3) at 80 °C to reduce remaining Fe(–Mn–Al) (hydr)oxides

The sum of the element solubilized in the steps 1 to 4 is referred to as extractable fraction in the following sections, while a residual fraction is defined as the difference between the total element concentration and the sum of the steps 1–4. Such residual fraction includes sulfide and silicate minerals (Dold 2003).

### Column leaching experiments

#### Column setup

**Fig. 1:**
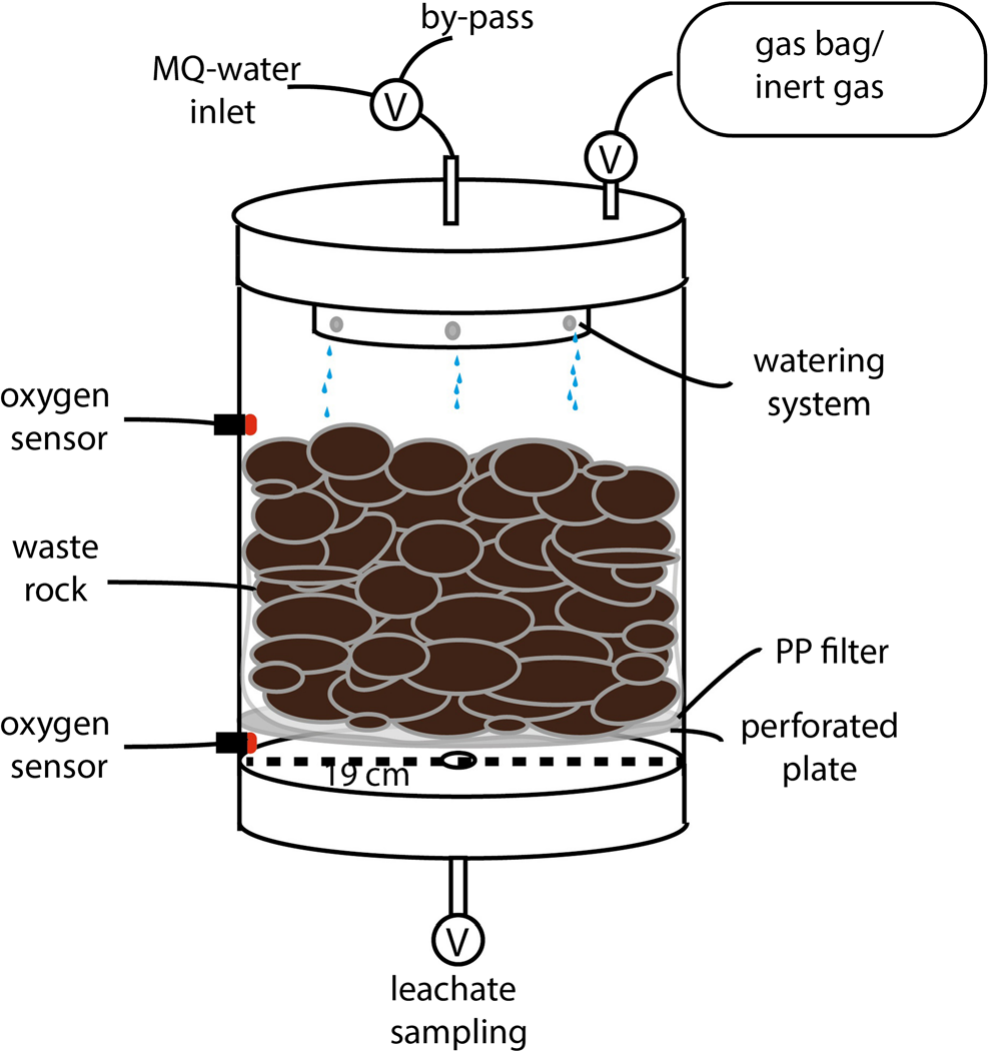
Experimental setup used for column leaching

Leaching experiments were carried out in free-draining leach columns constructed from Perspex glass tubes (diameter 19 cm), with caps made of polypropylene (Fig. 1). Waste rock (5.8 kg) sits on a perforated plate ca. 2.5 cm above the base of the column, with 105-μm-mesh polypropylene filter placed between the perforated plate and the waste rock. Prior to loading the waste rock into the columns, the column materials had been acid washed (1 M nitric acid, Merck® pro analysis) and thoroughly cleaned with MQ water. Uncrushed partially oxidized waste rock was used to avoid creation of fresh sulfide surfaces. Waste rock in the columns was wetted by feeding water through the six holes located in the cap of the column. Water was freely drained through the waste rock and collected at the column base, being no longer in contact with the waste rock. Leachate was sampled from the tap at the column base 4 h after wetting.

The column design does not follow any standard method as such does not exist for waste rock under anoxic conditions, but was inspired by the AMIRA free-draining column setup (AMIRA 2002) with significant modifications to allow operation under anoxic conditions. While standard humidity cell testing has been suggested to overestimate the reactivity compared with the field, such relationships are complex (Sapsford et al. 2009). It is uncertain how the current column design, differing from the standard design with respect to column dimensions, particle size, water volume, humidity, and oxygen input, affects the reactivity. The coarse particle size of the waste rock may rather lead to underestimation than overestimation of the reactivity, as it allows rapid percolation and results in short contact time between the waste rock and water.

Wetting and sampling of the leachate were done on a weekly basis, except for the first few weeks during which waste rock was wetted twice a week. After the first 10 cycles, when the leachate pH, redox potential (Eh), and electrical conductivity (EC) had stabilized, one of the columns was sealed from the atmosphere. The amount of wetting water used per cycle was 430 mL, expect for 600 mL used in the first cycle. This matches the future precipitation rates in northern Sweden, predicted to increase according to future climate change scenarios from the Swedish Meteorological Office (SMHI 2015), assuming each leaching cycle to represent 1 week during a year.

#### Oxygen measurements

The amount of oxygen transported to the column dissolved in the ingoing water corresponds to ca. 0.1 mmol O_2_ per cycle, thereby being fairly similar to a well-functioning dry cover system (ca. 0.5 mmol O_2_ per cycle, or 1 mol O_2_/m^2^ per year; Öhlander et al. 2012). Oxygen concentrations in the columns were continuously measured using O_2_-sensor spots (PreSens, Precision Sensing, Gmbh) attached to the columns at two different levels and calibrated against fully air-saturated (100 sat-%) and O_2_-free water (0 sat-%). The O_2_-sensor data are only considered semiquantitative because humidity variations in the columns were not measured but are known to have a large impact on the O_2_ saturation, and only a limited number of calibrations could be carried out during the experiment. During the first 12 months of leaching, no effort was made to remove O_2_ from the MQ water used to irrigate the column sealed from the atmosphere. In order to minimize contamination by O_2_, wetting and leachate sampling were done with the aid of an Ar gas and a filtration equipment was directly connected to the outlet. From the leaching cycle 51 onwards, the in-going MQ water was degassed by bubbling an N_2_ gas through the water for 20 min before wetting and leachate sampling were done under Ar gas flow, visually controlled by gas bubble generation from a discharge tube from the inlet water bypass.

#### Leachate analyses

Leachate mass was determined by weight, and leachate pH, oxidation–reduction potential (ORP), and electric conductivity (EC) were measured immediately upon sampling in the unfiltered leachate. The electrodes used were a glass electrode (SenTix® 82) for pH, Pt electrode (SenTix® ORP) for ORP, and TetraCon® 925 for EC. The electrodes were calibrated and checked against standard pH buffers, and EC and ORP standard solutions, respectively. Redox potential (Eh) with respect to a standard hydrogen electrode (SHE) was calculated from the ORP readings. For determination of dissolved solute concentrations, leachate was filtered through 0.2-μm cellulose nitrate filters (Whatman). The filters had been cleaned with acetic acid (5%) and thoroughly rinsed and soaked in MQ water prior to sampling. Anion samples (F, Cl, SO_4_) were not further treated. Samples for cation and Fe redox species determinations were preserved with nitric (1 mL 65% Merck Suprapur HNO_3_ per 100 mL sample) and hydrochloric (0.5 mL 30% Merck Suprapur HCl per 100 mL sample), respectively. The samples were stored in dark and cold (4 °C) until analyses. Additionally, selected samples of unfiltered leachate were collected for the determination of total element concentrations.

Over 70 major, minor, and trace elements were analyzed in selected leachate samples by quantitative screening analyses using inductively coupled plasma sector field mass spectrometry (ICP-SFMS) at the accredited ALS Scandinavia in Luleå, Sweden. The anion samples were analyzed for F and Cl using ion chromatography (IC) and potentiometric titrations at the ALS Czech Republic. Sulfate was analyzed by several methods, including gravimetry (ALS Czech Republic), turbidimetry (Luleå University of Technology, method U.S. Environmental Protection Agency 1986), and calculated from the total S concentrations determined reported from the ICP-SFMS. The determination of Fe total and Fe(II) concentrations was carried out with the ferrozine method (To et al. 1999) in filtered and acidified leachate samples at Luleå University of Technology. The possible changes upon sample storage and the potential errors introduced upon dilution were tested systematically, in addition to the comparison of the ferrozine-determined Fe_tot_ to that determined by the ICP-SFMS method.

### Geochemical modeling and calculations

Geochemical calculations, including aqueous ionic activities and species distribution, and mineral saturation state, were carried out using PHREEQC version 3.3.2 (Parkhurst and Appelo 1999) using the *wateq4f.dat* thermodynamic database (Ball and Nordstrom 1991). Only samples with full chemical analyses were considered in the calculations. The samples had an ion balance within ± 14%, with an average of 1.4% and a standard deviation of 6.6%. For mineral saturation state and redox calculations involving Fe(III) minerals, the wateq4f.dat database was updated with thermodynamic data from the literature. Solubility constants for freshly precipitated ferrihydrite, goethite, and jarosite were those reported in the Wateq4f database (Ball and Nordstrom 1991). The solubility constants of the more ordered (two- and six-line) ferrihydrites were those compiled and reported by Stefánsson (2007). For schwertmannite, the solubility constants from Majzlan et al. (2004), originating from Bigham et al. (1996), Yu et al. (1999), and Kawano and Tomita (2001), were considered.

The rate of solute release was estimated in terms of average release rate, moles of solute per kilogram of waste rock per cycle, after cycle 30. Moreover, the accumulated mass released from the waste rock to the leachate during the whole experiment was calculated based on the measured solute concentrations and leachate mass. As only selected leachate samples were analyzed for their chemical composition, the concentrations in the remaining samples were estimated by assuming linear concentration changes between the samples. Therefore, the total mass release is only an estimation and associated with uncertainties, but allows a comparison with the total element composition and the sequential extraction data of the waste rock.

## Results and discussions

### Waste rock geochemical and mineralogical characteristics

The chemical composition of the waste rock prior to its leaching in the present experiments is reported in Table 1. The waste rock is characterized by high S (20%) and Fe (18%) concentrations, with other major rock-forming elements including Si (19%), Al (6.5%), K (1.3%), and minor Ca, Mg, Na, and K. Sulfur is predominantly found as sulfide (99% of total S; Table 1), and together with total Fe concentration allows estimation of a pyrite content of 38 wt%. The low inorganic C content (0.06% TS) implies very low calcite content (ca. 0.5%). The chemical composition of waste rock thus reflects the previously reported primary mineralogy with pyrite and quartz dominating, along with traces of muscovite, chlorite, and calcite (Nyström et al. 2017). Sulfides other than pyrite identified in the waste rock include chalcopyrite, bournonite, sphalerite, and arsenopyrite (Nyström et al. 2017). Leaching of the waste rock under unsaturated conditions has previously been reported to produce a highly acidic leachate characterized by very high concentrations Fe, S, and many metal(loid)s, particularly those of As, Cu, Pb, Sb, and Zn (Alakangas et al. 2013; Nyström et al. 2019). In addition to the listed sulfide minerals, these metal(loid)s are also commonly contained in pyrite (Abraitis et al. 2004). Moreover, carbonates and silicates dissolve under acidic conditions, releasing associated elements to the leachate. Note that while the total concentrations of As and Sb in the waste rock are clearly elevated compared with average crustal composition (Table 1), the concentrations of Cu and Pb are low and that of Zn is only slightly elevated despite their high concentrations in the leachate. With the exception of As, the total concentrations of these metals are well below the Swedish guideline values for contaminated land (Swedish Environmental Protection Agency (SEPA) 2016).

**Table 1:** Total concentration of selected elements in the waste rock. Target values for contaminated land by SEPA ( 2016) are given for comparison

		Waste rock^a^	SEPA ( 2016)^b^
Element	Unit	Concentration	Target values
S^c^	%	20.2 (0.16)	
Fe	%	18	
Si	%	19	
Al	%	6.5	
Ca	%	0.80	
Mg	%	0.62	
Na^d^	%	0.38	
K	%	1.3	
Mn	%	0.014	
F^d^	%	0.13	
Cl^d^	%	0.01	
C^e^	%	0.11 (0.07)	
As	mg/kg	171	10/25
Ba	mg/kg	237	200/300
Cd	mg/kg	0.45	0.8/12
Co	mg/kg	5.0	15/35
Cu	mg/kg	13	80/200
Hg	mg/kg	12	0.25/2.5
Ni	mg/kg	13	40/120
Pb	mg/kg	16	50/400
Sb	mg/kg	15	12/30
Zn	mg/kg	115	250/500

^a^Data from sodium peroxide fusion unless otherwise stated

^b^Target values for both vulnerable and less vulnerable land use are given

^c^Total concentrations from sodium peroxide fusion and Leco furnace. Sulfate-S given in parenthesis

^d^Data from the lithium borate fusion on another subsample

^e^Data from Leco furnace on another subsample. Carbonate-C given in parenthesis

**Fig. 2:**
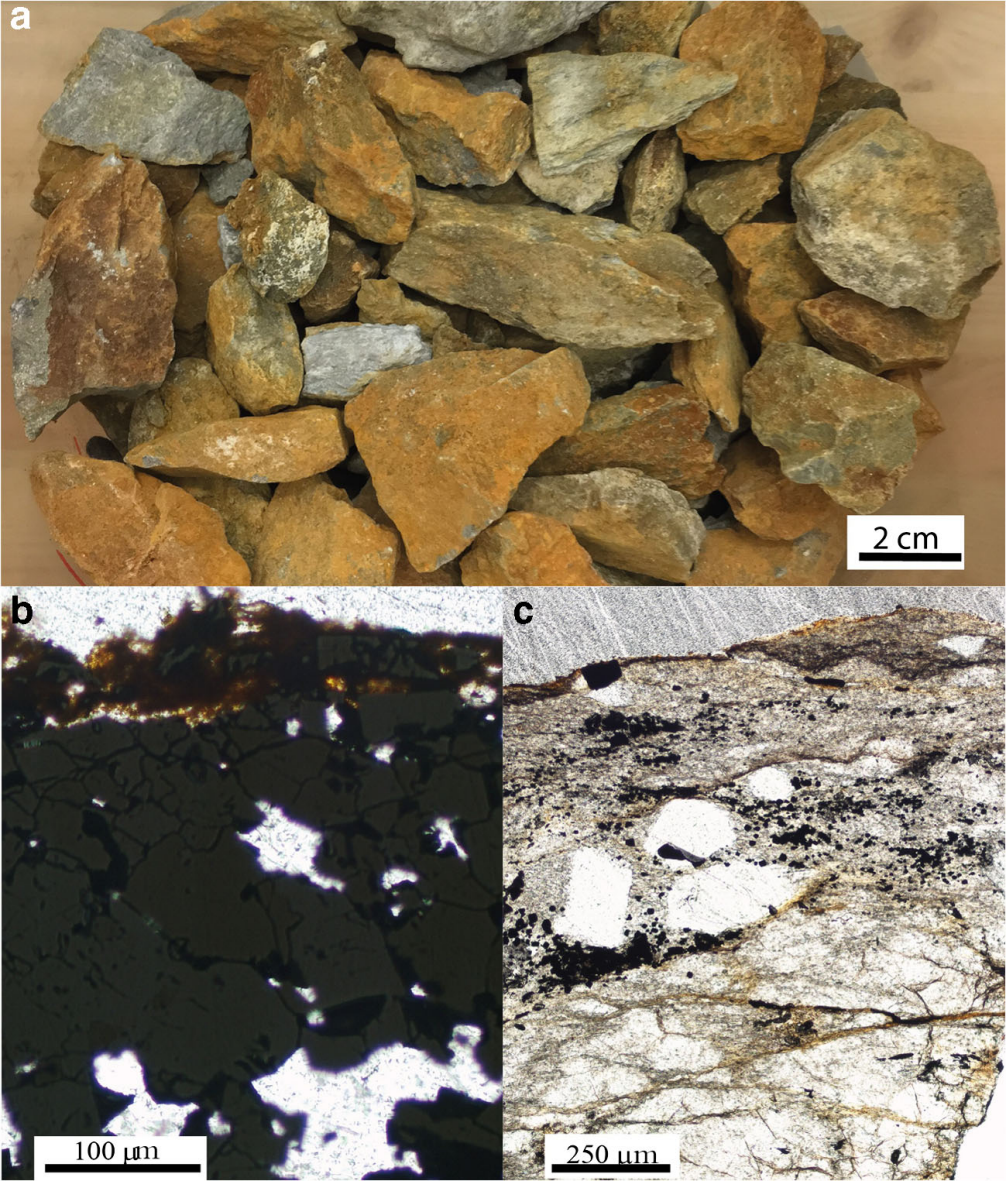
**a** Partially oxidized waste rock in the experimental column. **b** Optical microscopic image in transmitted light mode of pyrite-rich waste rock particle; note the rim of secondary minerals in the upper part of the image. **c** Optical microscopic image in transmitted light mode of silicate-rich waste rock particle

**Fig. 3:**
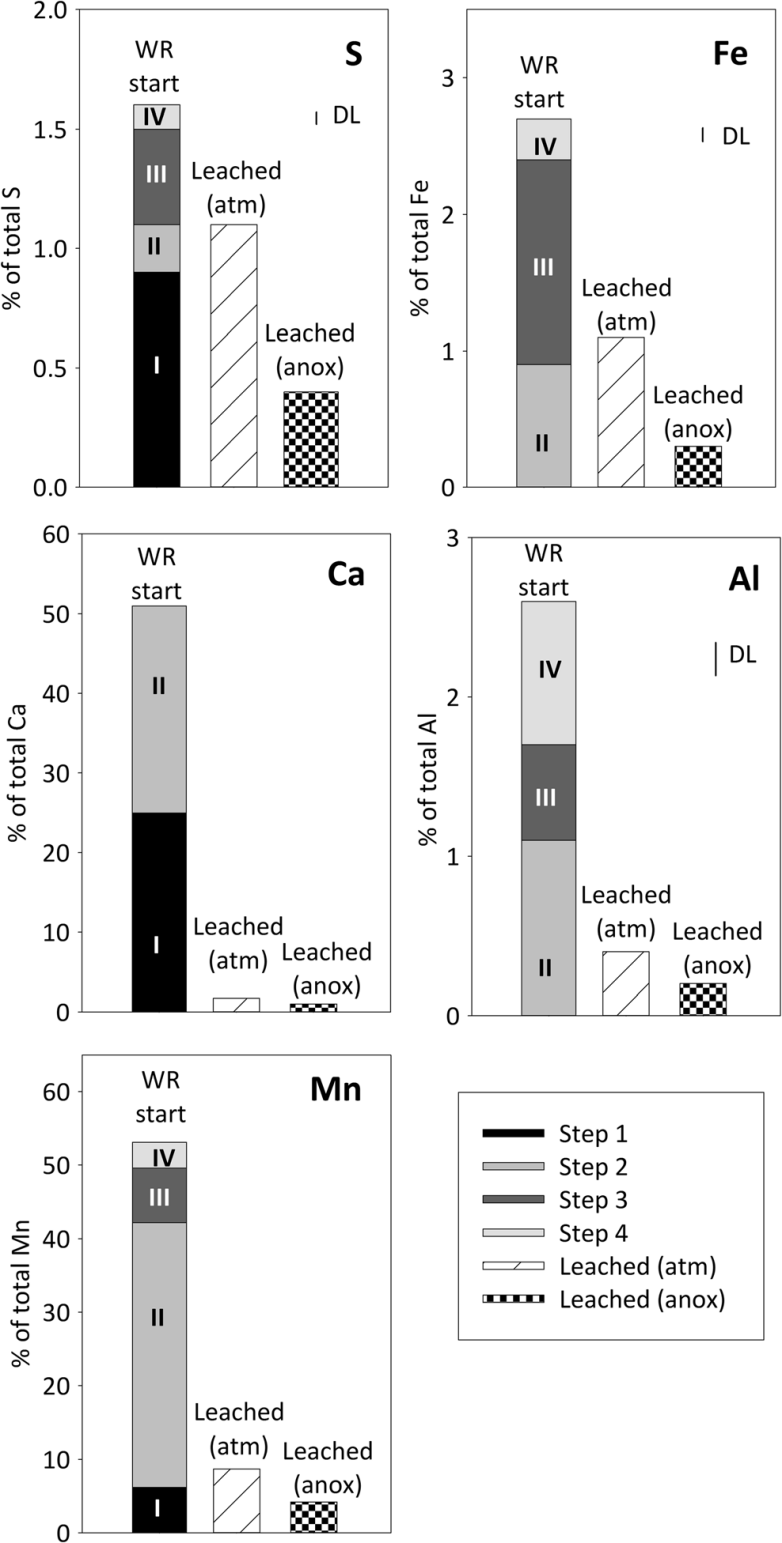
Sequential extraction data showing the association of S, Fe, Al, Ca, and Mn with the waste rock at the start (“WR start”). Also shown is the total mass leached during the whole experiment under the two conditions (“leach atm,” “leach anox”) (see “Geochemical modeling and calculations”). The units are percent of the total element in the waste rock and the total leached mass is compared to the total element concentration in the waste rock at the start. The small line labeled “DL” indicates the detection limit for each element, and values below detection limit are not plotted

Upon oxidative weathering over 6 years, secondary minerals may have accumulated in the waste rock. Waste rock particles at the start were coated with secondary mineral precipitates (Fig. 2a). Optical microscopy of selected waste rock particles shows silicate-rich waste rock particles stained with reddish–brown color and relatively unaltered pyrite-rich particles, typically surrounded by a thin reddish–brown layer (Fig. 2b, c). The element distribution between the primary and secondary minerals, as well as the type of secondary minerals, can be estimated from the sequential extractions and shows only a limited mass accumulation of secondary minerals. The common secondary minerals in the mine environment include (hydr)oxides and oxyhydroxysulfates of Fe and Al (e.g., Bigham and Nordstrom 2000). The sequential extraction data, however, shows that for Fe, S, and Al, the residual fraction consisting of sulfides and silicate minerals dominated in the partially oxidized waste rock at the start of the leaching experiments (Fig. 3). Less than 1.6% of total S and < 3% of total Fe and Al were extractable and therefore associated with the secondary minerals. Dissolution of clays and carbonates can also have contributed to the extractable amount (Dold 2003; see “Secondary minerals and metal(loid) association”). Dissolution of calcite is indicated by the high fraction of extractable Ca solubilized in step 2 that is in excellent agreement with the amount of calcite estimated based on the determined inorganic carbon content.

**Fig. 4:**
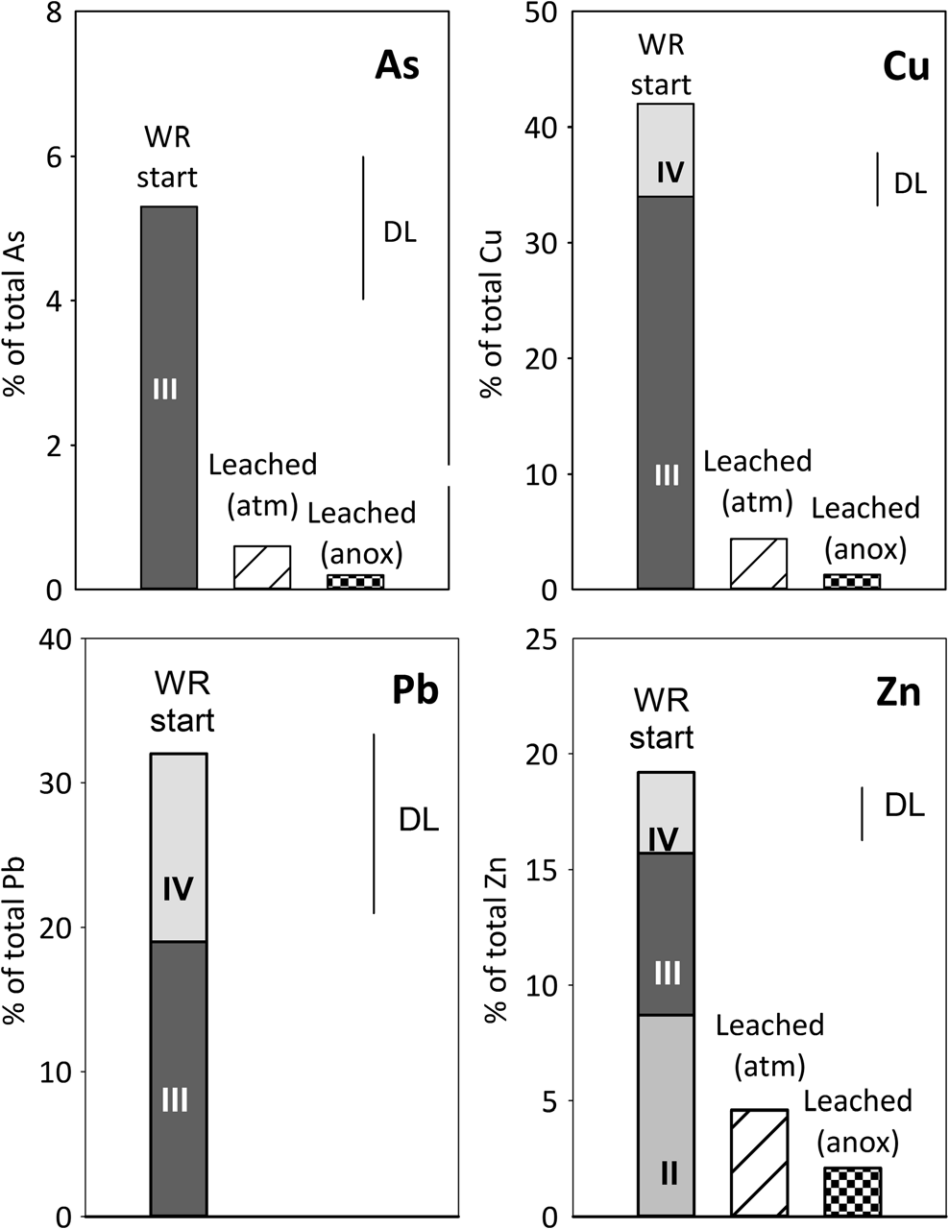
Sequential extraction data showing the association of As, Cu, Pb, and Zn with the waste rock at the start (“WR start”). Also shown is the estimated total mass of each element leached during the whole experiment under the two conditions (“leach atm,” “leach anox”) (see “Geochemical modeling and calculations”). Symbols and units are as those in Fig. 3

The metal(loid)s released to leachate upon oxidation of sulfide minerals are either transported away or sequestered by precipitation or co-precipitation to secondary minerals or by sorption processes on either secondary or primary minerals. The fraction of As, Cu, Pb, and Zn solubilized in the extraction steps 1–4, thereby associated with the waste rock by sorption, ion exchange, or secondary minerals prior to leaching experiments, varies over a large range from ca. 5% (for As) to over 40% (for Cu) (Fig. 4). This implies that for these elements, the residual sulfide/silicate fraction still is a predominant host of these elements in the partially oxidized waste rock.

It may be that the surface area of the large, centimeter-scale particles limited the mass of secondary minerals in the waste rock. Moreover, the acid pH conditions that had formed upon oxidation of the waste rock do not favor extensive accumulation of secondary minerals, but instead high mobility of S and Fe and possibly even dissolution of previously formed Fe(III)-containing minerals (e.g., Bigham and Nordstrom 2000). Note that percolating water in the first place interacts with the particle surfaces coated with secondary minerals, rather than with the inner, less reacted parts included in the analyses of pulverized samples.

### Secondary minerals and metal(loid) association

The secondary minerals identified using XRD, Raman, and SEM–EDS in the waste rock at the start of the experiments include gypsum, schwertmannite, ferrihydrite, and goethite. The sequential extraction data is in overall fair agreement with the observed mineralogy (Fig. 3).

Water-soluble minerals and salts, including gypsum, dissolve in step 1. Ca and S were the major elements solubilized in step 1, and the molar ratio Ca/S 0.89 extracted was close to that of gypsum (Ca/S = 1). No Fe or other metal(loid)s, with the exception of Mn, were detected in step 1. It is possible that other water-soluble phases, or alternatively dried pore water, were also present in the waste rock at low concentrations below the detection limited, as indicated by the elevated metal concentrations during the initial 1–3 leaching cycles (“Column experiments”).

Step 2 not only solubilizes weakly adsorbed and exchangeable forms but also dissolves carbonates and clays (Dold 2003). In addition to secondary minerals, primarily Fe(III) phases, other minerals such as quartz, plagioclase, and kaolinite are capable of adsorbing metals over a large range of pH levels, due to zero point of charge in the range of 2–4.6 (Stumm 1992). A significant fraction of the extractable Ca, Fe, Al, and some S was solubilized in this step. The association of Ca with this step (Fig. 3) is in excellent agreement with the measured inorganic carbon content, indicating dissolution of calcite. The solubilization of K and Al may arise from the dissolution of clays (Dold 2003), such as kaolinite, observed as a weathering product associated with the waste rock (Nyström, unpublished data). The minor and trace elements associated with this fraction include Mn and Zn (Figs. 3 and 4), but with the available data, it is not possible to more specifically assess the association mechanism.

Extraction step 3 solubilized most of the extractable Fe and S in the waste rock, in good agreement with the observation of schwertmannite and ferrihydrite by mineralogical methods. However, the molar ratio of Fe/S in schwertmannite (4.5–8) and ferrihydrite (no structural S) is clearly higher than the solubilized Fe/S ratio (2). Solubilization of Al and K in step 3 suggests that other minerals, such as jarosite (K,Na,H_3_O)Fe_3_(OH)_6_(SO_4_)_2_, alunite (KAl_3_(SO_4_)_2_(OH)_6_), or basaluminite (Al_4_(SO_4_)(OH)_10_ × 4H_2_O), also dissolved. These minerals commonly occur in a mine environment (Bigham and Nordstrom 2000), although these were not detected by mineralogical methods in the present waste rock. In agreement with the observation of goethite and ferrihydrite (six-line) by mineralogical methods, some Fe also solubilized in the extraction step 4. Some Al, S, and Mg were additionally solubilized in step 4, suggesting that also some other minerals were also dissolving. The minerals expected to dissolve in this step include jarosite and hematite (Dold 2003). In addition to Al-sulfoxyhydroxide phases listed above, Al along with Mn are commonly incorporated into Fe(III) (hydr)oxides. Acid oxalate has been shown to dissolve some silicates as well (Arshad et al. 1972), providing a possible explanation of solubilization of Mg.

The reducible fractions (steps 3 and 4) were the predominant hosts of extractable As, Cu, and Pb and also contained an important fraction of the extractable Zn and Mn. Arsenic was only detected in step 3, representing easily reducible phases such as poorly ordered ferrihydrite and schwertmannite. Copper, Mn, Pb, and Zn were, at least to some extent, also associated with the step 4 dissolving goethite, more ordered ferrihydrites, and jarosite. The association of metal(loid)s including As, Cu, and Pb, as well as Mn and Zn, with schwertmannite, ferrihydrites, and goethite is well established. Arsenic forms an oxyanion which tends to adsorb onto positively charged Fe(III) mineral surfaces at an acid pH and has a particularly high affinity to schwertmannite due to the formation of inner-sphere complexes (e.g., Regenspurg and Pfeiffer 2004; Acero et al. 2006; Nagano et al. 2011). In general, cation-forming metals, such as Cu, Mn, and Zn, are adsorbed to a lesser extent at a low pH, due to less favorable electrostatic attractions (Lee et al. 2002; Sidenko and Sherriff 2005; Acero et al. 2006; Nagano et al. 2011). However, adsorption of many cationic metals is enhanced in the presence of sulfate (Webster et al. 1998; Swedlund and Webster 2000; Swedlund et al. 2003). Studies on pure HFO phases, or mixtures thereof as typical for natural HFO, have shown preference of Cd and Zn for Fe(III)-sulfate phases and Pb for Fe(III) (hydr)oxides (ferrihydrite), while Cu association both into crystalline phases such as goethite, and Fe(III)-sulfates (Webster et al. 1998; Sidenko and Sherriff 2005). However, partially oxidized waste rock represents a complex mineralogical mixture, and the application of the studies on pure HFO is not straightforward.

The freshly formed poorly crystalline HFO schwertmannite and ferrihydrites tend to transform into more crystalline phases, mostly goethite, upon aging (e.g., Bigham and Nordstrom 2000; Jönsson et al. 2005; Kumpulainen et al. 2008). Similar change with goethite as a product has also been observed under changing redox conditions to the more reducing environment (Burton et al. 2008; Pedersen et al. 2005, 2006). The release of some, but in most cases only a fraction, of the associated metal(loid)s to the solution has been observed upon such transformations (Schroth and Parnell Jr 2005; Acero et al. 2006). However, such mineralogical transformations and the consequent changes in the metal(loid) association are poorly understood under conditions relevant to waste rock dumps.

The metal(loid)s most prone to remobilization under declining oxygen conditions with the sequential are those associated with the extraction step 3. The more resistant and crystalline phases extracted in step 4 require strongly reducing conditions. Therefore, As, Cu, and Pb, together with Mn and Zn, are expected to mobilize under anoxic conditions, along with and major elements associated with these fractions, namely Fe, S, and Al. Note that also decrease in pH concentration leads to the dissolution of minerals solubilized in steps 3 and 4 as their solubility is strongly dependent on pH. Additionally, Mn, Zn, Al, and Fe associated with step 2 may become remobilized under acid conditions, but also in response to changing solution composition. An overall improvement of water quality is, however, expected in a long term due to the limited oxidation of sulfides under anoxic conditions and decreased dissolution of gangue minerals (Al-silicates, calcite) and more favorable conditions for the adsorption of cationic metals under less acidic conditions.

### Column experiments

The extent and the rate at which the different reactions will proceed, and therefore the leaching behavior and water quality at mine closure, are difficult to predict based on solid phase characterization only. Column leaching experiments provide insight into the water quality expected upon cover application and to what extent the metal(loid)s become mobilized under changing chemical conditions. The partially oxidized waste rock originated from pilot-scale experimental leaching cells, collected after 5 years of leaching and having produced leachate with very high concentrations of S, Fe, As, Cu, S, Pb, and Zn, with the concentration levels depending on the water saturation and experimental conditions (Alakangas et al. 2013).

The oxidation of pyrite, the main sulfide in the waste rock, has been described by several authors previously (e.g., Sapsford et al. 2009; Maest and Nordstrom 2017) and can be summarized by Reactions (1)–(3):

1 $$ {\mathrm{Fe}\mathrm{S}}_2\left(\mathrm{s}\right)+{\mathrm{H}}_2\mathrm{O}+3.{5\mathrm{O}}_2={\mathrm{Fe}}^{2+}+{2\mathrm{SO}}_42--+{2\mathrm{H}}^{+} $$

2 $$ {\mathrm{Fe}}^{2+}+0.25{\mathrm{O}}_2+{\mathrm{H}}^{+}={\mathrm{Fe}}^{3+}+0.{5\mathrm{H}}_2\mathrm{O} $$

3 $$ {\mathrm{Fe}\mathrm{S}}_2\left(\mathrm{s}\right)+14{\mathrm{Fe}}^{3+}+{8\mathrm{H}}_2\mathrm{O}=15{\mathrm{Fe}}^{2+}+{2\mathrm{SO}}_42--+16{\mathrm{H}}^{+} $$

Reaction (1) describes pyrite oxidation by oxygen releasing sulfate and Fe(II), with consequent oxidation of Fe(II) by oxygen to Fe(III) according to Reaction (2), and participation of the formed Fe(III), an effective oxidant, in pyrite oxidation (Reaction (3)). Reaction (3) is considered the main pyrite oxidation pathway at a low pH, and the rate-limiting step in the propagation of pyrite oxidation under acid conditions has been suggested to be the oxidation of ferrous to ferric iron, i.e., Reaction (2) (Singer and Stumm 1970). In an acid mine environment, microorganisms capable of oxidation of Fe(II) are, however, ubiquitous and low pH allows for high Fe solubility and co-existence of both Fe(II) and Fe(III). Under these conditions, sulfide oxidation typically accelerates upon exponential growth of Fe-oxidizing microbes (e.g., Yu et al. 2001; Sapsford et al. 2009). Such acceleration of sulfide oxidation had occurred upon the previous testing of the waste rock in unsaturated pilot-scale cells and has been observed at ca. 30 weeks in laboratory-scale experiments (Alakangas et al. 2013; Nyström et al. 2019).

**Fig. 5:**
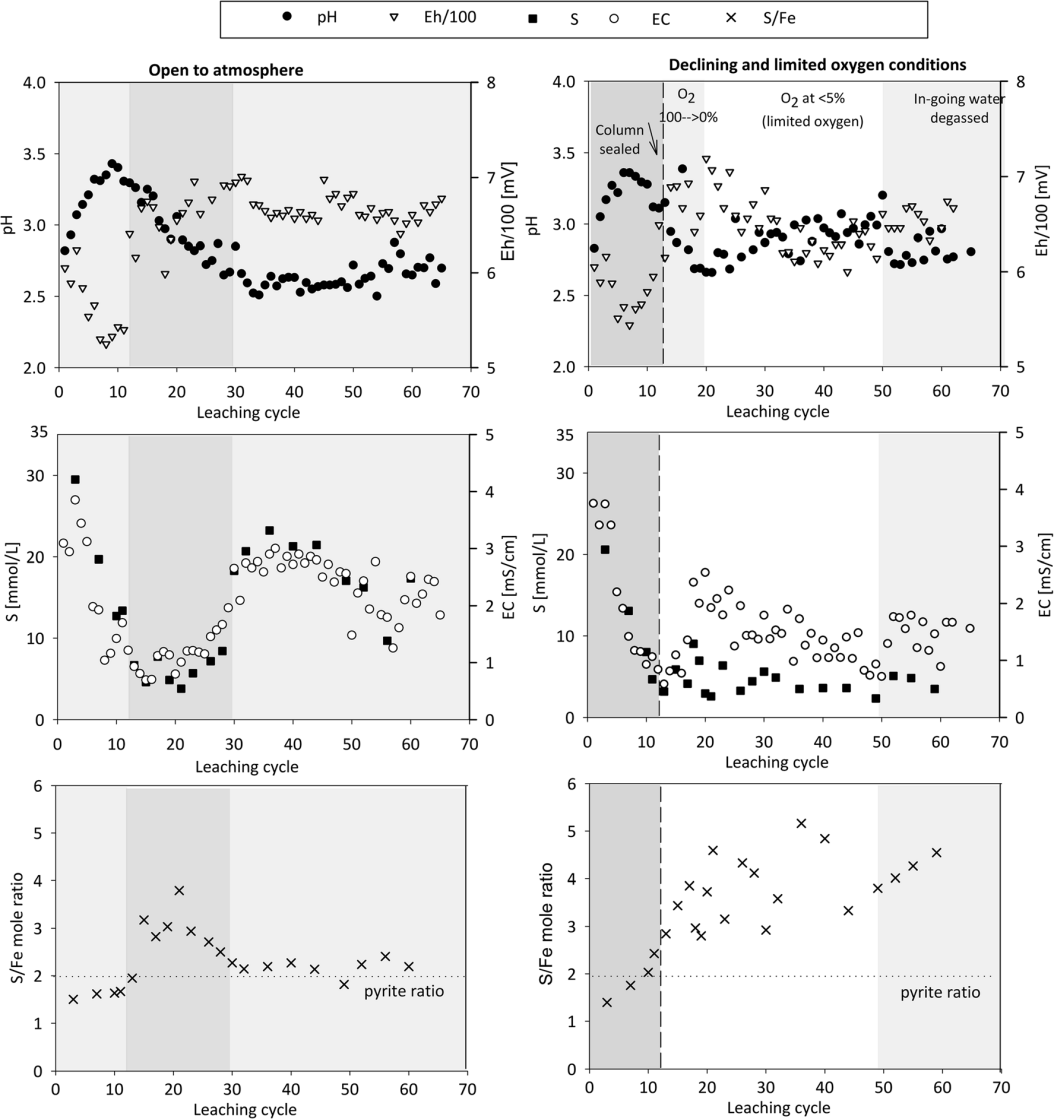
Evolution of leachate pH, Eh, EC, and SO_4_ concentrations and S/Fe molar ratio formed upon leaching of highly sulfidic waste rock under atmospheric conditions (**a**–**c**) and under limited and declining oxygen conditions (**d**–**f**). The different background colors refer to different processes thought to be dominating the leachate chemistry and the observed oxygen conditions in the sealed column according to Fig. 6

**Fig. 6:**
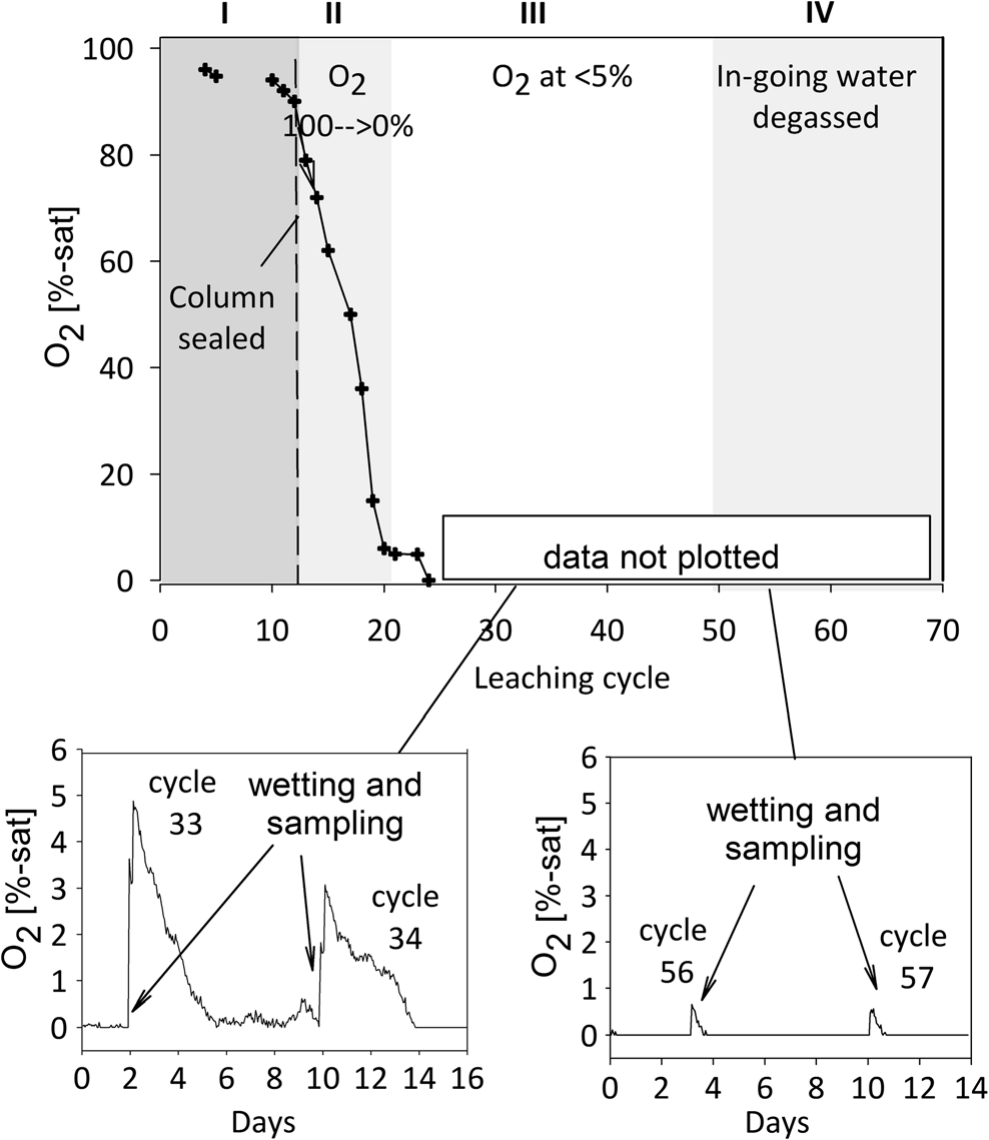
Declining oxygen concentrations in the column sealed from the atmosphere. The dotted line indicates sealing of the column, while roman letters indicate the oxygen conditions in the column: I, initial leaching under atmospheric conditions; II, declining oxygen conditions from 100 to 0 %-sat; III, limited oxygen/anoxic conditions, with small amounts of oxygen in the same range as expected in case of a well-functioning cover system introduced during wetting and sampling; and IV, further declining oxygen conditions when degassing of the ingoing water was initiated. The small figures show the disappearance of oxygen within a few days

**Fig. 7:**
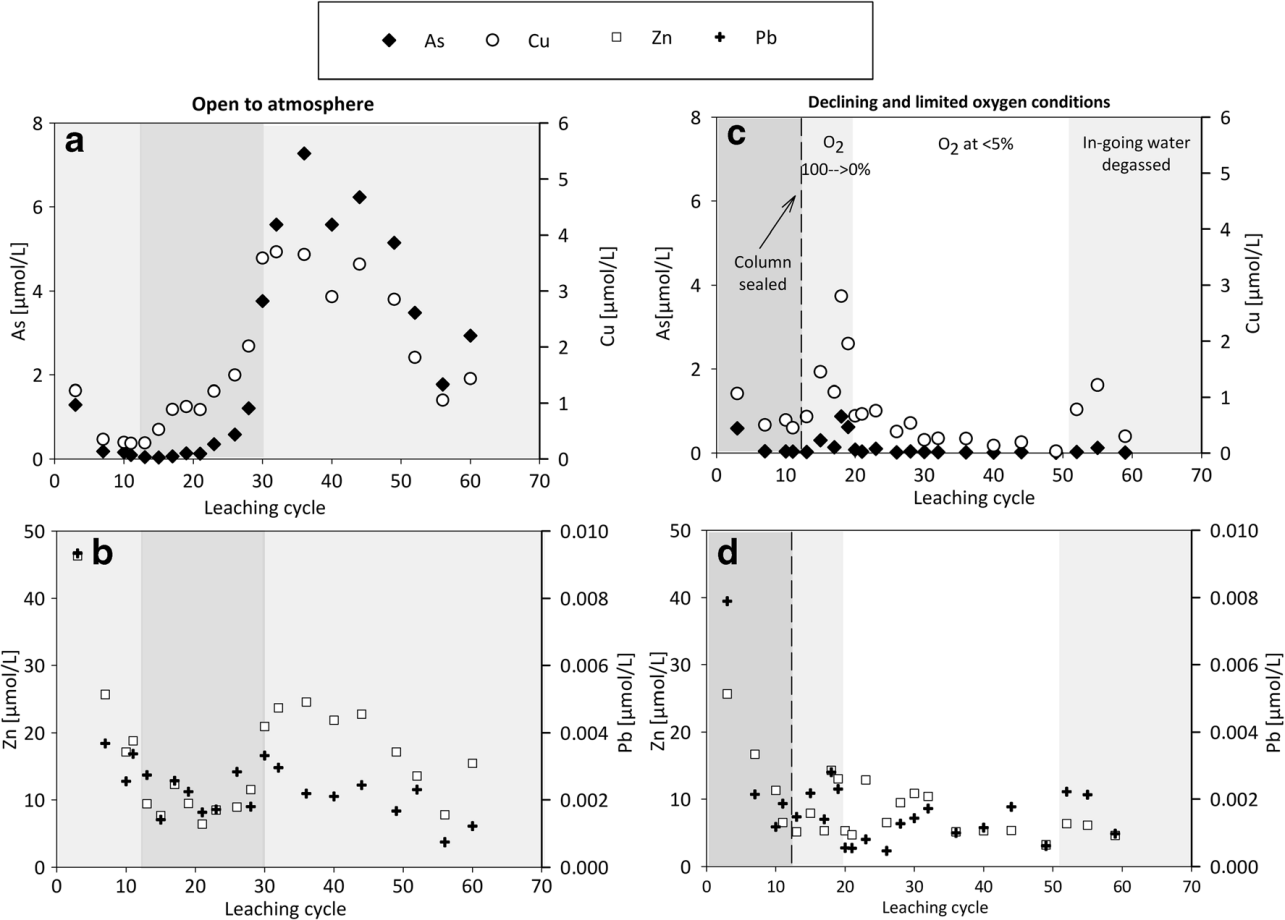
Concentrations of selected metal(loid)s in leachate produced from partially oxidized highly sulfidic waste rock open to the atmosphere (**a**, **b**) and during declining oxygen conditions (**c**, **d**). Note the different *y*-axis scales

The initial leachates formed upon re-wetting and leaching of the waste rock in the present experiments show highly acidic pH, elevated EC, and SO_4_ and Fe concentrations (Fig. 5). The oxygen conditions referred to in Fig. 5 are shown in Fig. 6. The evolution of leachate composition shows a pattern that is typical for the initial portion of leaching of partially oxidized sulfidic mine waste. It is characterized by initially elevated solute concentrations due to so-called early flush, i.e., dissolution of soluble salts and dried pore water upon re-wetting of the surfaces, followed by a period of relatively low concentrations prior to the start and acceleration of sulfide oxidation (e.g., Sapsford et al. 2009; Maest and Nordstrom 2017). In addition to initial low pH (2.8), high EC (3.8–4.0 mS/cm), and elevated major element (S, Fe, Ca, Al) concentrations, several minor and trace metal(loid)s were solubilized at high concentrations in the early flush (Figs. 5 and 7). The early flush is followed by an increase in pH (to 3.4) and a decrease in EC (≤ 1 mS/cm) and solute concentrations until cycle 10 (Figs. 5 and 7). Until cycle 11, both columns were leached under atmospheric conditions, after which the other column was sealed from the atmosphere. The continued leaching of waste rock under atmospheric concentrations represents a situation where the waste rock is allowed to oxidize without covering, while the sealed column presents coverage of the waste rock, resulting in declining and limited oxygen availability.

#### Continued leaching under atmospheric conditions

After the first flush, leachate formed under atmospheric conditions shows decreasing pH, while EC increases (Fig. 5). Between cycles 12 and 25, the leachate Fe/S mole ratio varies in the range of 2.5–4, but towards cycle 30 approaches the ratio of 2 as in pyrite. The concentrations of most solutes, including Fe, SO_4_, and many metal(loid)s, show an overall increase and reach their maximum between cycles 30 and 50, corresponding to ca. 9–12 months of leaching (Figs. 5 and 7). Accordingly, decrease in the leachate pH, increase in Fe concentrations, shifts to the predominance of Fe(III) (data not shown), and Fe/S ratios approaching those in pyrite are observed between cycles 20 and 30, indicating re-acceleration of pyrite oxidation and consequent acidity production between cycles 20 and 30. This is probably due to the drying of the waste rock and consequent re-start of the pyrite oxidation and microbial activity.

The solute concentrations do not maintain maximum level but show a decreasing trend and some scatter with time (Figs. 5 and 7). A decrease in laboratory air temperature by ca. 4–5 °C occurred after cycle 40 at the start of the winter season with fluctuation in the air temperature between 16 and 21 °C during the winter months and provided one explanation for the decreasing concentrations. Decreasing concentrations with time, however, are often observed in humidity cell testing (e.g., Sapsford et al. 2009; Maest and Nordstrom 2017) and interpreted to reflect slower sulfide oxidation rates and reaction rate limited by the available surface area.

In addition to SO_4_ and Fe, the other major elements in the leachate included Mg, Al, F, and Ca in decreasing order of abundance (Table 2). Concentrations of Mn, Si, K, and Na were typically below 0.1 mmol/L. The release of these major and minor elements reflects weathering of gangue minerals, including silicates but based on sequential extractions also dissolution of carbonate (for Ca, possibly Mn) and (hydr)oxides (for Al, Mn) may contribute (Fig. 3). Arsenic, Cu, and Zn are present in the leachate at very high concentrations according to the Swedish EPA (SEPA 2000) surface water classification for biological effects. In contrast to previous studies of the waste rock in question (Alakangas et al. 2013), the concentrations of Pb are very low (Table 2).

**Table 2:** Comparison of the average release rate after cycle 30, and classification according to SEPA ( 2000)

Average release rate^a^		
	Atmospheric	Limited oxygen
*[mmol per kg of waste rock]*		
SO4	1.34	0.28
Fe	0.62	0.07
Ca	0.05	0.03
Mg	0.19	0.06
K	0.0003	0.0002
Al	0.17	0.06
F	0.09	0.04
Na	0.001	0.001
*[μmol per kg of waste rock]*		
As	**0.33**	*0.002c*
Cd	**0.002**	0.0006
Co	0.06	0.01
Cu	**0.19**	***0.03d***
Mn	3.4	1.2
Ni	*0.010*	*0.002*
Th	0.030	0.002
Pb	*0.0001*	*0.0001*
Zn	**1.34**	**0.42**
pH	2.64 (s.u.)	2.90 (s.u.)
EC	2.4 (mS/cm)	1.3 (mS/cm)
Eh	669 (mV)	641 (mV)

^a^Average release rate per leaching cycle calculated after cycle 30

^b^SEPA (2000) classification of Cu, Zn, Cd, Cb, Cr, Ni and As concentrations in surface water: low concentration, moderately high concentration, high concentration, very high concentration

^c^Ranges from low to moderately high according to SEPA (2000) classification

^d^Ranges from moderately high to very high according to SEPA (2000) classification

**Fig. 8:**
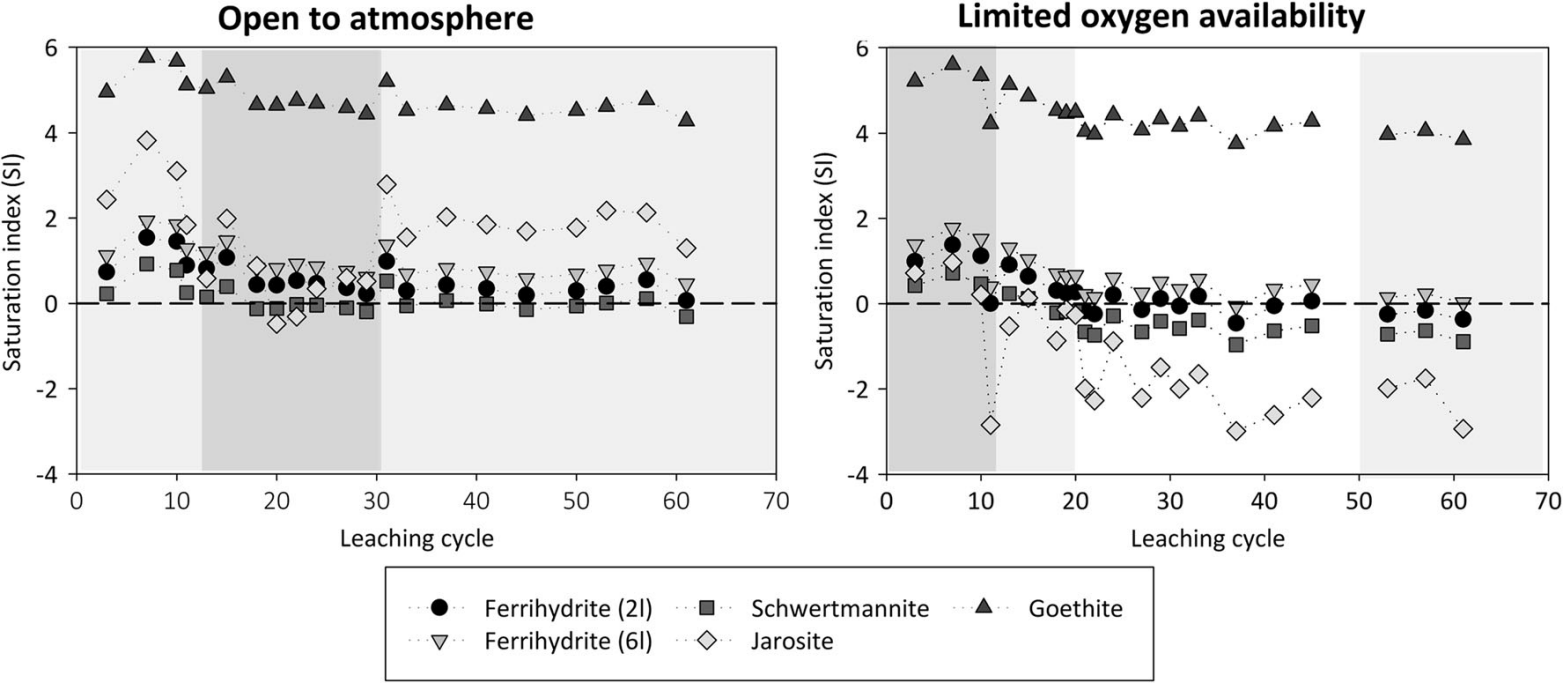
Evolution of the saturation index calculated for the leachate with respect to various Fe(III) minerals

The maximum concentrations of As, Cu, Mn, Pb, and Zn coincide with the lowest leachate pH, the maximum concentrations of Fe and S, and the Fe/S ratio corresponding to that in pyrite (Figs. 5 and 7). Thus, pyrite oxidation is considered to be the main process releasing these metal(loid)s under atmospheric conditions, either due to direct association of these elements with the oxidizing sulfides or due to the dissolution of gangue minerals under acid conditions resulting from the pyrite oxidation. Although masked with increasing concentrations towards cycle 30, an additional, rather minor local increase in the concentrations of most metal(loid)s can also be observed during cycles 12–25. This coincides with decreasing leachate pH and S/Fe ratio (ca. 2.5–4) differing from that in pyrite (2) and observed during the remaining experiment. Metal(loid)s were probably additionally released from another source, either primary or secondary. Dissolution of secondary Fe(III) minerals such as jarosite and schwertmannite is indicated by the coincident decrease in their saturation index, while during the remaining experiment, saturated indexes indicated saturation or supersaturation of these and other Fe(III) minerals (Fig. 8). Alternatively, decreasing pH may have resulted in desorption of cationic metals due to less favorable electrostatic attractions (Lee et al. 2002; Sidenko and Sherriff 2005; Nagano et al. 2011).

#### Declining and limited oxygen concentration

To study the leaching behavior of partially oxidized waste rock upon cover application, the availability of O_2_ to the column was limited by sealing the column from the atmosphere at cycle 11. Initially, an inert N_2_ gas was used to decrease the oxygen concentrations below 60 sat-% (cycle 16). After this, oxygen declined from 100 to 0 sat-% within 10 cycles of the sealing without further aid (Fig. 6). With the declining oxygen conditions, a temporal decrease in leachate pH and an increase in leachate EC occurred (Fig. 5). The increase in leachate EC, as a general indicator of leachate quality, reflects the peak concentrations of all solutes around cycle 20 (Figs. 5 and 7). The concentrations of several metal(loid)s, including Al, Mn, As, Cu, and Zn, peaked only over a few cycles and then decreased, reaching a relatively low level.

During the following 12 months of leaching (until cycle 50), no effort was made to remove oxygen from the ingoing MQ water, and therefore, the oxygen dissolved in MQ water entered the column during each leaching cycle (“Column leaching experiments”; Fig. 6). The period between cycles 20 and 50, therefore, represents leaching of the waste rock under limited oxygen availability. Calculations based on the semiquantitative O_2_-sensor data indicate that ca. 0.5–1 mmol O_2_ entered the column per cycle. This is 5–10× higher than expected (“Column leaching experiments”) and thereby closely matches requirements for a cover system. The oxygen introduced at each leaching cycle was consumed within 1–2 days (Fig. 6), presumably due to sulfide oxidation (see “Column experiments”). Leachate pH slightly increased between cycles 20 and 50 but still remained highly acidic (pH ~ 3) (Fig. 5).

After 12 months of leaching (from cycle 50 onwards), degassing of the in-going water was initiated in order to minimize O_2_ ingress to the column. Still, traces of O_2_ (< 1 sat-%) were detected by the continuous measurements in association with wetting and sampling but disappeared within 1–2 days (Fig. 6). In response to such further decrease in oxygen conditions, leachate pH slightly decreased, EC increased, and a short temporal release of several solutes occurred again (Figs. 5 and 7).

**Fig. 9:**
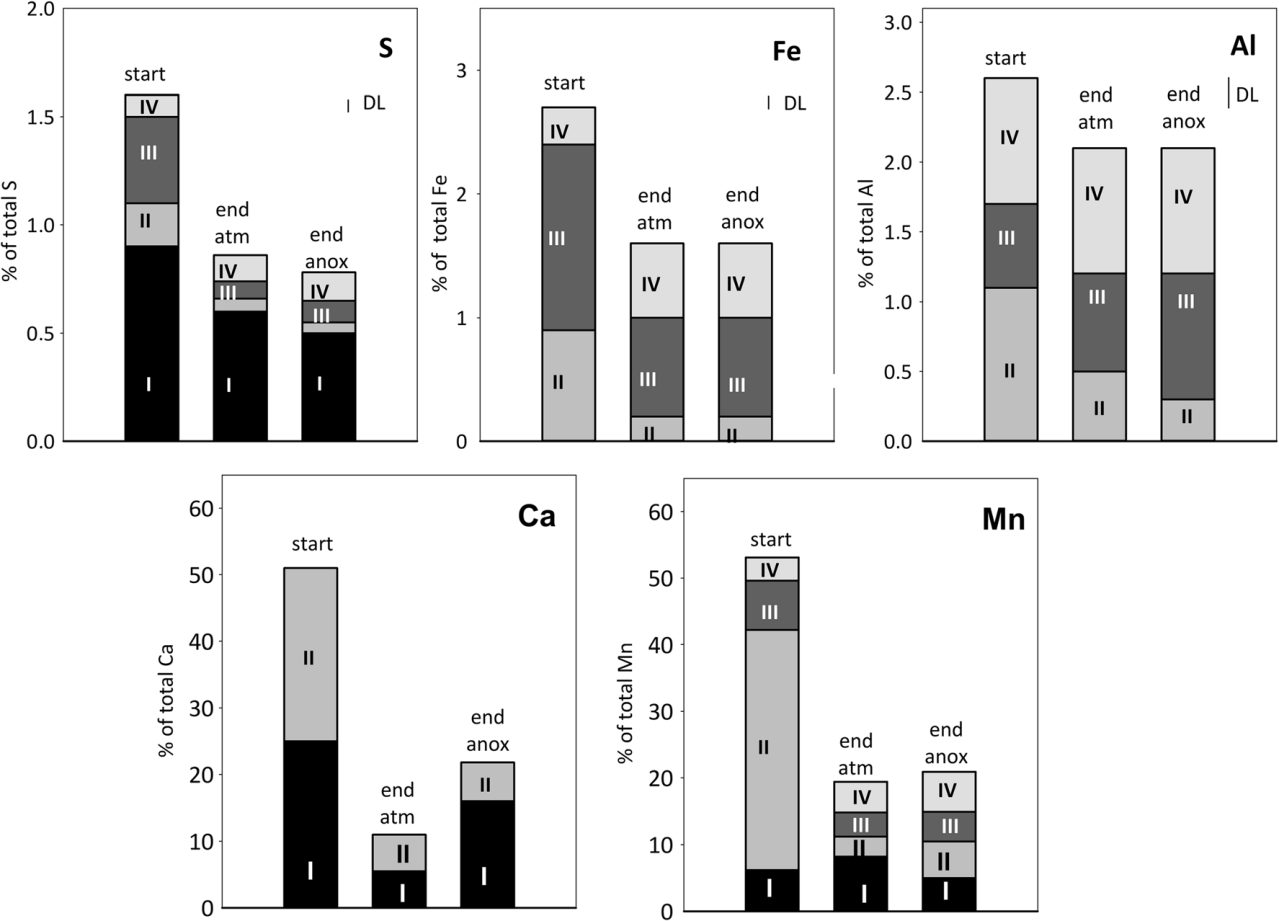
Sequential extraction data showing the association of S, Fe, Al, Ca, and Mn with the waste rock at the start (“start”) and end of the experiments under atmospheric (“end atm”) and limited oxygen conditions (“end anox”). The units are percent of the total element in the waste rock. The small line labeled “DL” indicates the detection limit for each element, and values below detection limit are not plotted. Note that sequential extraction data between the different samples may not be directly comparable

Sulfate concentrations in the leachate were clearly lower under limited oxygen conditions compared with atmospheric conditions (Table 2; Fig. 5). Moreover, Fe concentrations, and particularly those of Fe(III), were lowered. This is reflected in the higher S/Fe ratio (2.5–4) in leachate, different from that in pyrite and observed under atmospheric O_2_ conditions. Sulfide oxidation does not re-accelerate similarly to the atmospheric conditions, in good agreement with the low Fe_total_ and Fe(III) concentrations. However, the quick disappearance (ca. 1 day) of the O_2_ introduced at each cycle shows ongoing oxidation (Fig. 6). The sulfate release from the experiment after cycle 30 is in fair agreement with what could be produced via sulfide oxidation considering the maximum estimate for the O_2_. Clearly, some sulfate originated from sulfide oxidation, but a simple mass balance consideration shows that S leached out during the experiment could also be explained by solubilization from secondary phases (Figs. 3 and 9) including schwertmannite and gypsum, as well as exchangeable S associated with the extraction step 2 (Fig. 3; “Waste rock geochemical and mineralogical characteristics”). Indeed, saturation state calculations of Fe(III) minerals show that with experimental progress, the leachate became progressively undersaturated with respect to Fe(III)-sulfate minerals schwertmannite and jarosite, while Fe(III) hydroxides remain close to saturation or supersaturated (Fig. 8). The dissolution of Fe(III)-S phases releases both Fe and S, but consequent immobilization of Fe by precipitation of Fe(III) hydroxides would result in increasing leachate S/Fe ratio. This is in agreement with the limited release of Fe during the experiment (Fig. 3) and increased relative importance of more crystalline Fe-hydroxide phase during the experiment (extraction step 4; Fig. 9).

The dissolution of Fe-S phase is expected to lead to remobilization of the associated metal(loid)s, which at least to some extent may then become incorporated into newly forming Fe(III) hydroxides, thereby resulting in peaking metal(loid) concentrations. Arsenic and Cu concentrations peak prominently, followed by decreasing concentrations to a low background level within a few cycles (Fig. 7). Other metals including Al, Mn, and Zn also peak, but to a lesser extent and the following concentration decrease not being as clear as for As and Cu. The peak concentrations were typically clearly lower than the concentrations observed under atmospheric conditions and during the initial re-wetting of the waste rock. Overall, considering the average release rate calculated after cycle 30, the release of most major and minor rock-forming elements as well as many potentially harmful metal(loid)s, in particular of As, decreased clearly compared with atmospheric conditions (Table 2).

**Fig. 10:**
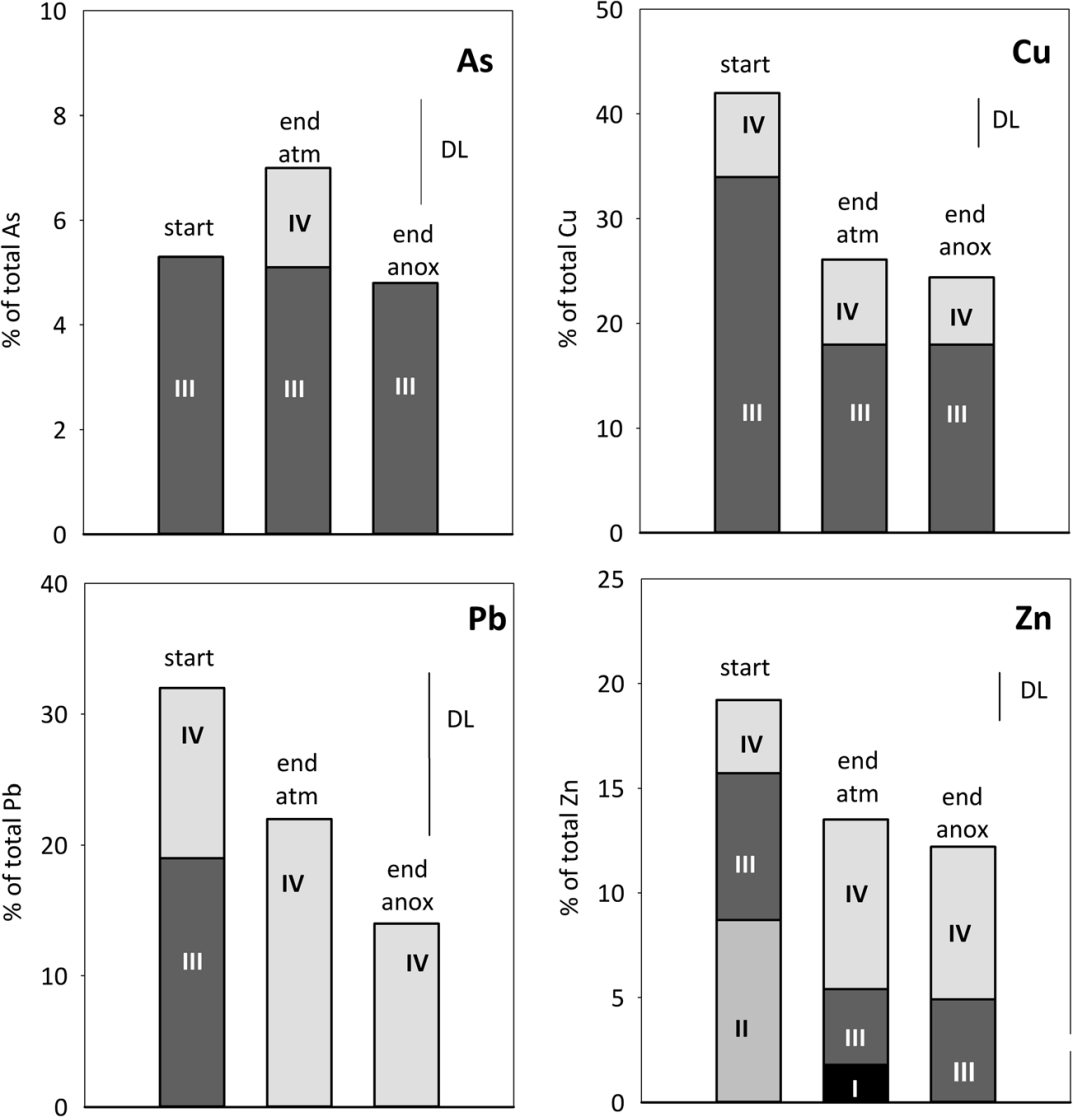
Sequential extraction data showing the association of As, Cu, Pb, and Zn with the waste rock at the start and end of the experiments. Symbols and units as those in Fig. 9

Arsenic is the element of major concern associated with the waste rock, and its concentrations in the leachate reached a very low to a low level (SEPA 2000) under limited oxygen conditions, with the exception of the peak concentration associated with the declining oxygen conditions after cycle 50. The most important reason behind the low As concentrations is considered to be the limited oxidation of pyrite and arsenopyrite, the predominant hosts of As in the waste rock according to the sequential extraction (Fig. 4; “Waste rock geochemical and mineralogical characteristics”). Arsenic is the element that peaks prominently upon declining oxygen conditions (Fig. 7). However, the peak concentrations during declining conditions were, however, an order of magnitude lower than those reached under atmospheric conditions and decreased to a low background level within a few cycles (Fig. 7). The peak As concentrations observed during declining oxygen conditions were probably caused by the remobilization of As associated with extraction step 3, including schwertmannite, suggested to dissolve during declining and limited oxygen condition (Fig. 8; “Secondary minerals and metal(loid) association”). The quick decrease following the peak As concentrations indicates the uptake of As to the new solid Fe(III) phase suggested to form based on saturation state calculations and observed at the bottom of the column at the end of the experiment. As an oxyanion, As has high affinity to Fe(III) minerals even under acid conditions (e.g., Regenspurg and Pfeiffer 2004; Nagano et al. 2011). Overall, only a small fraction of secondarily associated As was mobilized and therefore As remains associated with the extraction step 3 in the waste rock after the experiment (Fig. 10), in addition to the residual (sulfide) fraction, the predominant As host.

Of the cationic metals, Cu showed somewhat similar behavior to As, with significantly lower overall concentrations under limited oxygen concentrations compared with atmospheric conditions, and temporally elevated concentrations associated with declining oxygen conditions. Unlike As, the maximum Cu concentrations reached under the two experimental conditions were similar. Similarly to As, Cu was associated predominantly with the reducible fraction (steps 3 and 4) in addition to residual/sulfide fraction, and its association with the solid phase did not change during the experiment (Fig. 10). In addition to limited sulfide oxidation, Cu was released from reducible fraction (extraction step 3). The differing behavior of As and Cu is probably due to the fact that Cu as a cationic metal only adsorbs to a limited extent under acid conditions, pH ~ 3, maintained throughout the experiment. Despite the decreased concentrations under limited oxygen conditions, Cu remained at a high to a very high level according to SEPA ( 2000) classification.

The maximum concentrations and release rate of Zn decreased less than those of Cu and As. Zinc concentrations also peaked during declining oxygen conditions, although not as clearly as those of As and Cu (Fig. 7). Despite the decreased concentrations under limited oxygen conditions, Zn remained at a high to a very high level according to SEPA ( 2000) classification. The reason for this is considered to be its remobilization from the exchangeable/carbonate fraction extracted at step 2, which, unlike in case of As and Cu, hosts significant Zn at the start of the experiment and clearly decreases during the experiment under both conditions (Fig. 10). This is in good agreement with the high mobility of this fraction, and the unfavorable sorption conditions or carbonate dissolution under acid pH, or release from exchangeable sites due to changing solution composition. It is possible that also the reducible fraction, sulfide oxidation, or acid-dissolution of silicates also contributed to the Zn release. Major and minor rock-forming elements Al and Mn show some similarities to Zn: concentrations were decreased to somewhat limited extent, and the importance of extraction step 2 (exchangeable/carbonate fraction), hosting significant fraction of extractable Al and Mn at the start, had clearly decreased at the end (Fig. 9).

Concentrations of Pb in the leachate produced in the present study (< 0.004 μmol/L) under both atmospheric and limited oxygen conditions are low compared with the concentrations reported for the leachate produced from the same waste rock previously (ca. 0.02–1 μmol/L; Alakangas et al. 2013), despite the similar concentrations reported in the solid phase. According to the sequential extraction, ca. 30% of Pb associated with the reducible fraction of the waste rock, although there is a large uncertainty associated with the analyses due to low concentrations. The possible reasons for the low mobility of Pb include exhaustion of the available Pb prior to the present experiment and/or the unavailability minerals hosting Pb, e.g., due to large particle size, its immediate uptake to stable secondary minerals, as well as the stability of the Pb-hosting secondary minerals. Even if some mobilization may have occurred upon declining oxygen conditions (Fig. 7), the concentrations in the leachate remain very low.

### Mass considerations and practical implications

High concentrations of metal(loid)s were released to the leachate under atmospheric conditions, even though the waste rock had previously been exposed to oxidation and consisted of relatively large particles used in the experiments. Despite the high concentrations of As, Fe, S, Cu, and Zn in the leachate produced upon leaching of the waste rock under atmospheric conditions, their release to the leachate corresponds to < 1% of the total element present in the waste rock for As, Fe, S and to 3–4% in case of Cu and Zn (Table 2). Assuming that the whole waste rock is reactive and the average release rate calculated for the experiment under atmospheric conditions, a rough estimation can be made that the contamination source will be available for several decades or over 100 years, highlighting the need for preventing sulfide oxidation. Note that the total concentrations of Cu and Zn in the waste were lower or just slightly elevated, respectively, compared with the average crustal composition and well below the general guideline values for contaminated land (SEPA 2016; Table 1). However, they were released to the leachate at high concentrations under both the atmospheric and limited oxygen conditions, demonstrating the risk associated with correctly identifying the contaminants of concern, and therefore the environmental risks, in the absence of long-term kinetic testing.

Only a fraction of the total amount of metal(loid)s associated with the reducible fractions became mobilized under limited oxygen conditions (Figs. 9 and 10). Moreover, some Fe, Al, and Mn were still present in the exchangeable/carbonate fraction and Mn also in the water-soluble phase after the experiment. In agreement with our observation regarding the importance of exchangeable fraction for divalent metals, sorption processes have been shown to be particularly important for metal release from waste rock with low acid-producing potential, with partially oxidized waste generating Ni at much higher rates than fresh waste rock samples (Plante et al. 2011). Based on the release rates observed under limited oxygen conditions in this study, it can be roughly estimated that ca. 1–3 years would be required to leach all the extractable As, Cu, and Zn present in the waste rock at the end of the experiment and assuming no further partitioning of metal(s) into this fraction. Much longer time, in the range of 10–25 years, is required for leaching the extractable Al, Fe, and Mn. The remobilization of elements from the reducible fractions, however, is expected to be highly dependent on the strength of reducing conditions. Such reducible fraction defined operationally by the sequential extractions, taking advantage of aggressive chemicals to quantitatively dissolve the reducible phases, may exhibit metastability under anoxic or mildly reducing conditions, such as those in the unsaturated column experiments or in the field. In the experiments, the measured Eh values did not fall below 600 mV despite the limited oxygen conditions, most likely due to the buffering by the Fe system, involving Fe(III)-hydroxide phases (Kaasalainen et al. 2019). It is uncertain how strongly reducing conditions will actually develop in soil-covered waste rock dump. As an example, while covering tailings with biosolids cover resulted in the reduction of goethite with consequent remobilization of associated As (Paktunc 2013), covering tailings with low organic cover did not, within 2 years, create conditions reducing enough to remobilize As from secondary minerals (DeSisto et al. 2017).

Acid pH conditions were still maintained after 1 year of leaching under limited oxygen conditions relevant to those for a well-functioning cover system, and pH-raising measures may need to be considered. An explanation for such low pH may be buffering by Fe(III) minerals, where the Fe system buffers not only leachate pH but also leachate Eh and sulfate concentrations (Kaasalainen et al. 2019). Such buffering action is also likely to continue as long as these phases are present in the system. Moreover, it is also possible that release of S, Fe, and Al, associated with the extraction step 2 (Fig. 9), produces acidity due to hydrolysis reactions. The overall effect of alkaline addition in order to increase the neutralization capacity of the waste on metal(loid) mobility is uncertain.

Comparing the time scale required to mobilize any extractable metal(loid)s with their release under atmospheric conditions (“Mass considerations and practical implications”), it is clear that the focus should be preventing sulfide oxidation in the waste rock. The high sulfide content of the waste rock is an important factor not only for the assessment of the future pollution potential but also for creating the acid pH conditions that have a major effect on secondary mineral stability and metal(loid) mobility, as well as acceleration of sulfide oxidation. Sulfide oxidation mobilizes various solutes and leads to accumulation of secondary mineral and metal(loid)s, the amount and location of such accumulation depending on the environmental conditions along the water transport path. The partial oxidation will, therefore, complicate the future remediation efforts by causing sustained acidic pH and elevated metal(loid) concentrations, such as demonstated here, and therefore require prolonged water treatment at the mine closure, along with a larger mass of water treatment waste to be handled. Alternative strategies include successive remediation, inhibition of sulfide oxidation, or a development of a technical solution of extract and collect metal(loid)s from the oidizing waste rock.

### Field applications

Extrapolation of these experimental results to field conditions is challenging due to the particle size effects and the different biogeochemical and hydrogeological conditions in the field. There are some important differences between the experimental approach and what is expected upon application of a dry cover. Limited oxygen conditions, relevant to those for a well-functioning cover system, prevailed in the sealed column after cycle 20 or so. In a waste rock dump, any oxygen transported through the cover is expected to be consumed by sulfide oxidation in the top part of a high column of waste rock, thereby hindering oxygen transport to the deeper parts of the dump. Therefore, the environmental conditions in the transport path of water in the dump vary and affect the leachate composition. In the experiments, however, such a process is limited by the small size and height of the column. Moreover, an actual dry cover will prevent not only the ingress of oxygen but also water percolation, thereby limiting contaminant transport. In the experiments, waste rock was leached under unsaturated conditions in order to allow sampling and analyses of leachate in order to study element release during declining oxygen conditions. Ultimately, water is required as the transport media for metal(loid)s, and in the absence of water, no transport of metals occurs, except with the water already present in the waste rock dump. Compared with the column experiment, the percolating water in the dump will be transported over longer distances, and therefore, the water/rock ratio and contact time will differ from those in the present study. Despite these differences, such column leaching approach provides useful insights into the leaching behavior of metal(loid)s under environmental conditions expected upon remediation. Sequential extractions, although providing quantitative information on element distribution in primary and secondary minerals, offer limited insights into leachate quality. Taking advantage of aggressive chemicals to quantitatively dissolve different minerals phases, sequential extractions tend to overestimate the amount of potentially mobilize elements compared with natural settings. Therefore, leaching tests as those presented here complement such data, giving more realistic insights into reaction rates and water quality upon mine closure.

## Conclusions

Metal(loid) mobility from partially oxidized sulfidic waste rock was studied under declining and limited oxygen conditions in unsaturated column experiments with the aim to understand water quality at mine closure upon cover application. The study leads to following conclusions:

- Partially oxidized highly sulfidic waste contains a limited amount of secondary minerals and produces highly acidic leachate with high concentrations metal(loid)s, highlighting the need for preventing sulfide oxidation.
- Total concentrations in the waste rock did not reveal which elements are of concern, and leaching tests are required to identify these.
- Although acid pH (~ 3) conditions were sustained, the quality of leachate produced under limited oxygen conditions relevant to those expected for a well-functional cover system was clearly improved compared to the leachate formed under atmospheric conditions.
- Concentrations of As decreased the most, followed by Fe and Cu, as well as Al, Zn, and Mn. The concentrations of Cu and Zn remain problematic.
- A short-term negative impact on water quality due to peaking metal(loid) concentrations, likely caused by dissolution Fe(III)-sulfate minerals, occurred coincidently with declining oxygen conditions but was only of short duration and concentration levels remained clearly lower, or in the similar range, as the concentrations observed under atmospheric conditions.
- The sustained low pH, most likely due to secondary mineral buffering, plays an important role for metal(loid) mobility, and release of cationic elements from the exchangeable fraction is one of the reasons behind the maintained elevated concentrations of at least Al, Mn, and Zn
- Only a fraction of secondary minerals and associated metal(loid)s was released during the leaching testing. Leaching tests, such as those presented here, complement sequential extractions and mineralogical analysis and give useful insights into reaction rates and leachate quality upon mine closure.
- Although prevention of sulfide oxidation remains the main focus of remediation of partially oxidized waste rock, accumulation of secondary minerals and adsorbed metal(loid)s complicate the future remediation by, e.g., sustaining acidic pH and buffering metal(loid) concentrations and pose difficulties for predicting future water quality.
